# Pioneering a New Frontier: Modeling the Epithelial-immune Cell Axis Using Human Intestinal Organoids

**DOI:** 10.1016/j.jcmgh.2025.101461

**Published:** 2025-01-23

**Authors:** Scott T. Magness

**Affiliations:** Lampe Joint Department of Biomedical Engineering, University of North Carolina at Chapel Hill and North Carolina State University, Chapel Hill, North Carolina; Department of Cell Biology and Physiology, Center for Gastrointestinal Biology and Disease, School of Medicine, University of North Carolina at Chapel Hill, Chapel Hill, North Carolina

The gastrointestinal tract is a complex ecosystem where epithelial and immune cells coexist and interact to maintain homeostasis while responding to external challenges. Understanding the epithelial-immune axis is essential for uncovering the mechanisms behind inflammatory bowel diseases, host-pathogen dynamics, and for developing advanced therapeutic strategies. In this issue of *Cellular and Molecular Gastroenterology and Hepatology*, Tominaga et al^1^ present a cutting-edge study describing the generation of human intestinal organoids (HIOs) that incorporate functional tissue-resident macrophages, all derived from human pluripotent stem cells (PSCs). The team’s innovative approach provides a robust platform to study the interplay between epithelial and immune cells and sets a new standard in organoid-based research.

Building on a rich foundation, co-culture systems combining epithelial and immune cells have evolved significantly over the years. Early studies, such as those using immortalized epithelial cell lines, such as T-84 and Caco-2, to assess T-cell and monocyte interactions, provided foundational insights into epithelial barrier function and immune modulation.^2^, ^3^, ^4^ More recently, Zachos et al shaped the field by demonstrating the potential of human intestinal enteroids, also defined as epithelial-only organoids, to recapitulate key aspects of the intestinal microenvironment.^5^^,^^6^ Their work revealed the ability of enteroids to model mucosal infections and highlighted critical immune-epithelial interactions. Tsuruta et al^7^ injected induced PSC-derived monocyte-like cells into the subepithelial space of apical-out HIOs, but these cells exhibited limited responses to inflammatory stimuli.^7^ To address the limitations of culture-only systems, work by Bouffi et al used humanized mice to develop immune tissues in transplanted HIOs.^8^ Together, these studies advanced our understanding of how epithelial cells respond to pathogens and immune signals^5^ and highlight the innovative strides in creating more comprehensive immune-epithelial models.

The study by Tominaga et al represents a significant advancement in modeling the epithelial-immune cell axis in culture. By deriving both epithelial and macrophage populations from human PSCs and co-culturing them within organoids containing mesenchyme, the authors create a physiologically relevant system that mimics the development and function of tissue-resident macrophages in the intestine. Central to their success was leveraging concepts from developmental biology to direct the differentiation of cells into specific lineages. By mimicking embryonic cues, the team was able to generate macrophages with transcriptional profiles resembling those of human fetal intestinal macrophages and integrate them into organoids.

There are a number of key findings described in this study. First, macrophages incorporated into HIOs persisted for up to 12 weeks in a humanized mouse model and displayed transcriptional signatures akin to human fetal intestinal macrophages. This longevity and functionality distinguish this model from previous studies. Second, HIO macrophages were capable of phagocytosing bacteria and producing inflammatory cytokines in response to lipopolysaccharide stimulation, demonstrating their utility in modeling immune responses and inflammatory conditions like inflammatory bowel diseases.^9^ Single-cell transcriptomic profiling further revealed that macrophages co-cultured with HIOs acquired a transcriptional signature resembling tissue-resident macrophages in the human fetal intestine. These cells also expressed chemokines and cytokines that suggested active participation in inflammatory responses. Importantly, the study uncovered crosstalk between immune cells and other mesenchymal populations. It was observed that exogenous macrophage colony-stimulating factor or granulocyte–macrophage colony-stimulating factor is not required to differentiate or maintain macrophages within HIOs and that macrophage colony-stimulating factor is provided by the organoid mesenchyme further underscoring the complexity of an immune-mesenchymal-epithelial axis. Finally, the ability of interleukin-10 to modulate macrophage responses highlights the potential of the model for studying regulatory pathways involved in inflammation resolution.

Although the study by Tominaga et al represents a leap forward, it also opens new opportunities for further exploration. For instance, the co-cultured macrophages, although closely resembling tissue-resident macrophages, reflect fetal transcriptional profiles and provide a starting point to investigate how these cells transition to adult-like functionality. Expanding the model to incorporate interactions with the broad diversity of immune sub-lineages could further enrich its ability to replicate the immune microenvironment. Additionally, studying the long-term effects of chronic inflammation or repeated insults could reveal new insights into disease progression and therapeutic responses. These opportunities underscore the potential of the culture system as a versatile and evolving platform for investigating complex immune-epithelial dynamics.

By integrating functional tissue-resident macrophages into HIOs, Tominaga et al have set a benchmark in organoid-based research. As this model is adopted and refined, it will undoubtedly yield transformative insights into the epithelial-immune interactions that govern gastrointestinal health and disease. By bridging the gap between epithelial and immune research, this study exemplifies the power of interdisciplinary approaches to advance our understanding of complex biological systems.
